# Supplementary material for: Unearthing a novel function of SRSF1 in binding and unfolding of RNA G-quadruplexes
Source: Nucleic Acids Res. 2024 Apr 3;52(8):4676–90. doi: 10.1093/nar/gkae213 (PMC11077049; doi:10.1093/nar/gkae213)
Supplement: gkae213_Supplemental_File [file gkae213_supplemental_file.pdf]

Unearthing a Novel Function of SRSF1 in Binding and Unfolding of RNA G-Quadruplexes

Naiduwadura Ivon Upekala De Silva, Nathan Lehman, Talia Fargason, Trenton Paul, Zihan Zhang, Jun Zhang*

AUTHOR ADDRESS: Department of Chemistry, College of Arts and Sciences, University of Alabama at Birmingham, CH266, 901 14th Street South, Birmingham, AL, 35294-1240, USA

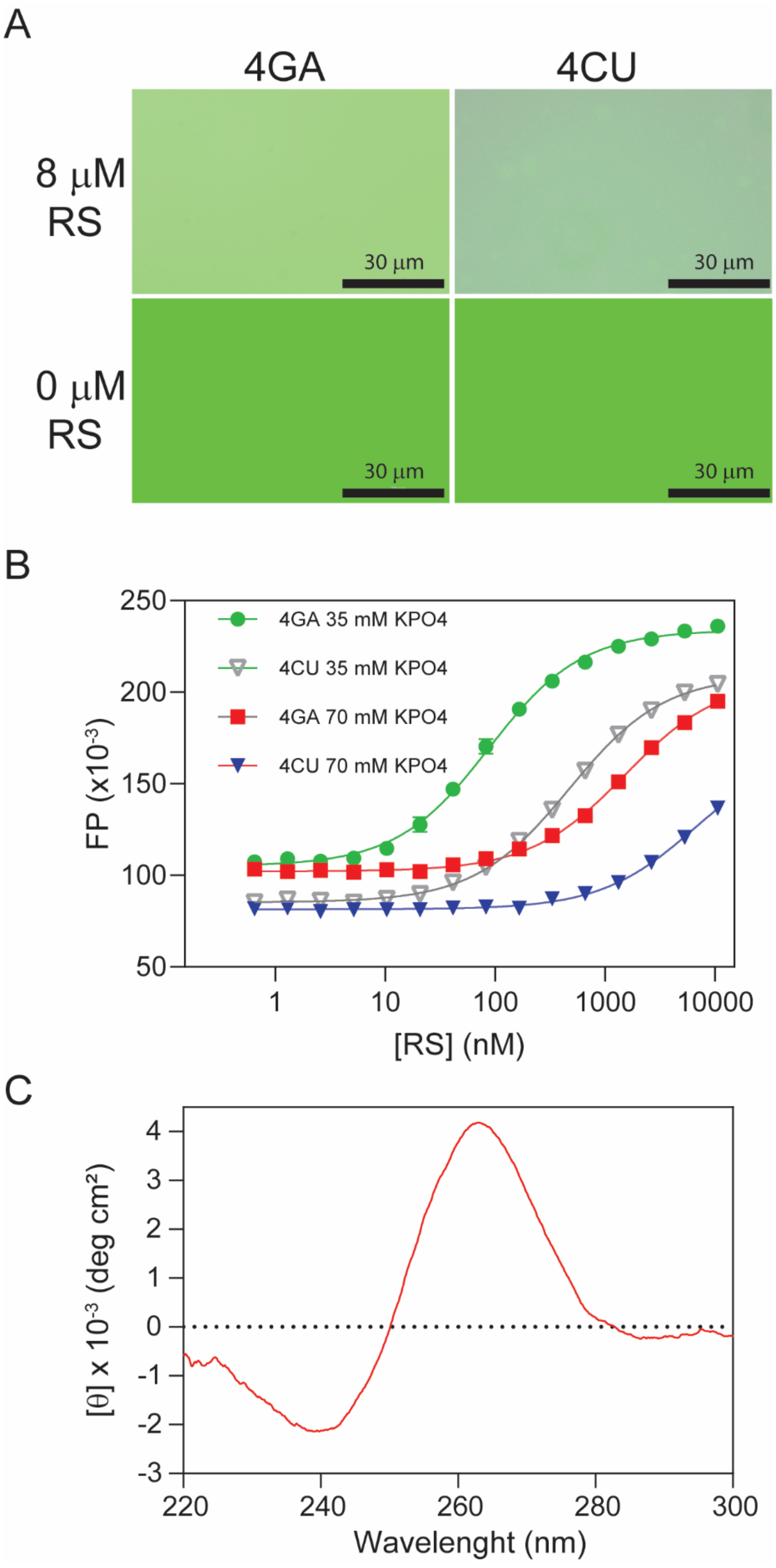
A
4GA
4CU
8 μM RS
0 μM RS
30 μm
30 μm
30 μm
30 μm
B
4GA 35 mM KPO4
4CU 35 mM KPO4
4GA 70 mM KPO4
4CU 70 mM KPO4
FP (x10-3)
250
200
150
100
50
1
10
100
1000
10000
[RS] (nM)
C
[θ] x 10-3 (deg cm²)
4
3
2
1
0
-1
-2
-3
220
240
260
280
300
Wavelenght (nm)

Figure S1: (A) RS does not form phase separation with 8-mer RNA in the FP binding buffer that contains 35 mM K_3PO_4, 2.5 mM NaCl, 15 mM HEPES pH 7.5, 0.015% TWEEN 20, and 0.075 mM TCEP. The images were recorded by a Cytation 5 plate reader. The blank bars indicate 30 µm. (B) Fluorescence polarization binding profiles for RS with 4GA and 4CU nucleotides in 35 and 70 mM K_3PO_4. RNA oligomers were labeled by fluorescein at the 5' end, and the fluorescent probe concentration was 10 nM for all binding assays. (C) Circular spectrum of 8G RNA. The spectrum was collected with 10 µM of RNA in 35 mM K_3PO_4, 2.5 mM NaCl, 15 mM HEPES pH 7.5, 0.015% TWEEN 20, 0.075 mM TCEP.

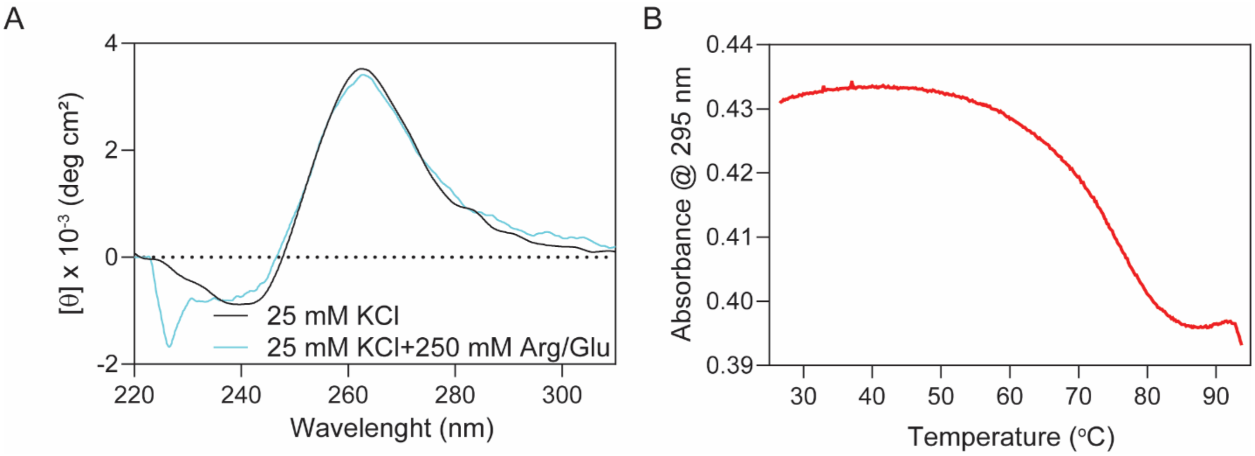

Figure S2: The ARPC2 GQ RNA sequence forms G-quadruplex. (A) Circular dichroism spectra of ARPC2 GQ in 25 mM KCl with and without 250 mM Arg/Glu. Addition of Arg/Glu does not change the secondary structure of ARPC2 GQ. Due to strong absorbance of Arg/Glu, the CD signal lower than 230 nm could not be accurately measured and therefore unreliable. (B) The UV melting curve of ARPC2 GQ. The data was collected at a concentration of 5 µM in a buffer containing 25 mM KCl, 0.25 M Arg/Glu, 20 mM Tris-HCl, pH 7.5, 0.1 mM EDTA, and 0.1 mM TCEP.

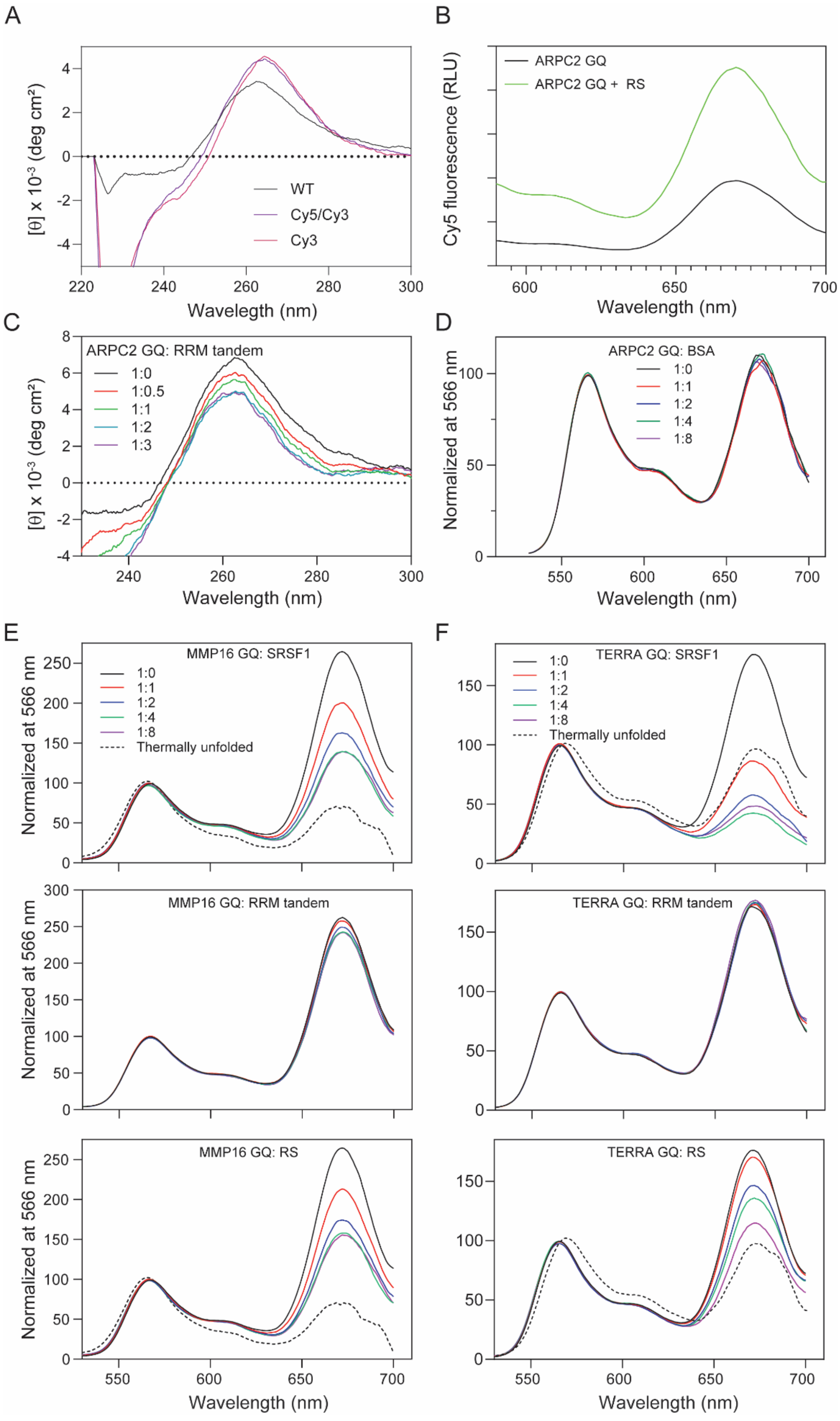

A
[θ] x 10-3 (deg cm²)
Wavelegth (nm)
WT
Cy5/Cy3
Cy3
B
Cy5 fluorescence (RLU)
ARPC2 GQ
ARPC2 GQ + RS
Wavelength (nm)
C
ARPC2 GQ: RRM tandem
1:0
1:0.5
1:1
1:2
1:3
[θ] x 10-3 (deg cm²)
Wavelength (nm)
D
ARPC2 GQ: BSA
1:0
1:1
1:2
1:4
1:8
Normalized at 566 nm
Wavelength (nm)
E
MMP16 GQ: SRSF1
1:0
1:1
1:2
1:4
1:8
Thermally unfolded
MMP16 GQ: RRM tandem
MMP16 GQ: RS
Normalized at 566 nm
Wavelength (nm)
F
TERRA GQ: SRSF1
Thermally unfolded
TERRA GQ: RRM tandem
TERRA GQ: RS
Wavelength (nm)

Figure S3: (A) CD spectra of fluorophore labeled ARPC2 GQ RNA. Cy5/Cy3-labeling does not affect the ARPC2 GQ structure. CD spectra were collected at 5 μM. (B) The Cy5 fluorescence of Cy5-ARPC2 GQ-Cy3 with and without 400 nM RS. The RNA concentration for apo RNA is 200 nM. The spectrum with 400 nM of RS (ARPC2 + RS) was normalized to the same concentration as the apo RNA. The samples were excited with a light at 500 nm. The Y-axis is in the relative light unit (RLU). (C) Circular dichroism of 5 μM of ARPC2 GQ titrated with different concentration of the RRM tandem. (D) Fluorescence spectra of 200 nM ARPC2 GQ titrated with different concentrations of BSA. The spectra were recorded with exciting light at 500 nm. All fluorescence and CD spectra were collected in 25 mM KCl, 0.25 M Arg/Glu, 20 mM Tris-HCl pH 7.5, 0.1 mM EDTA, 0.1 mM TCEP, and 0.02% TWEEN 20. The roles of SRSF1 domains in unfolding MMP16 (E) and TERRA (F) GQs.

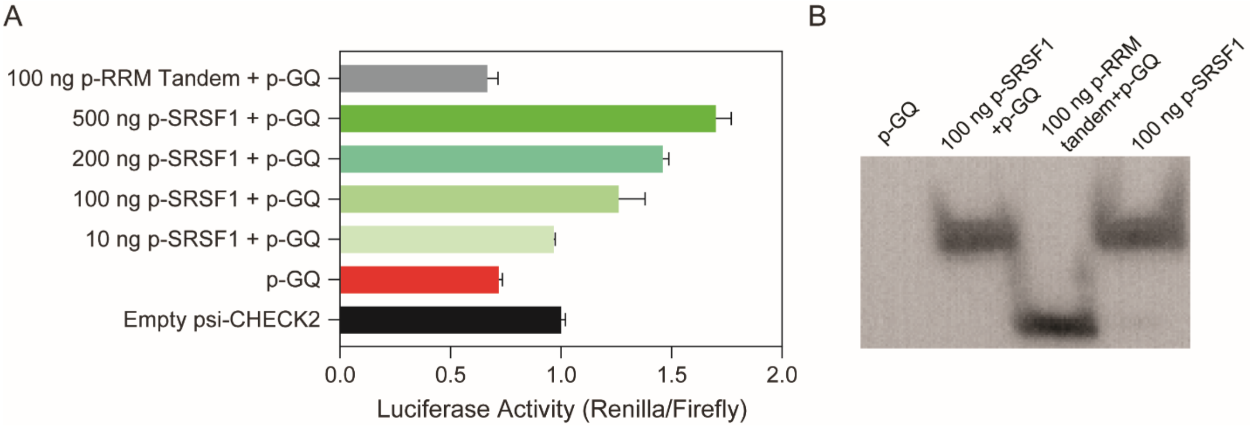

Figure S4: (A) Renilla and firefly luciferase activity ratio for HEK293 cells transfected by empty psi-CHECK2 (100 ng), or psi-CHECK2-GQ (100 ng), or co-transfected by psi-CHECK2-GQ and pcDNA4 plasmid harboring SRSF1 constructs. All assays were performed three times for error estimation. The luciferase luminescence was recorded by a Cytation 5 plate reader. (B) Confirmation of SRSF1 and the RRM tandem expression by western blot analysis. p-GQ: plasmid psi-CHECK2-ARPC2 GQ, p-SRSF1: plasmid pcDNA4-SRSF1, p-RRM: pcDNA4-SRSF1 RRM tandem.
